# Angiotensin receptor blockers for the treatment of covid-19: pragmatic, adaptive, multicentre, phase 3, randomised controlled trial

**DOI:** 10.1136/bmj-2022-072175

**Published:** 2022-11-16

**Authors:** Meg J Jardine, Sradha S Kotwal, Abhinav Bassi, Carinna Hockham, Mark Jones, Arlen Wilcox, Carol Pollock, Louise M Burrell, James McGree, Vinay Rathore, Christine R Jenkins, Lalit Gupta, Angus Ritchie, Ashpak Bangi, Sanjay D’Cruz, Andrew J McLachlan, Simon Finfer, Michelle M Cummins, Thomas Snelling, Vivekanand Jha, CLARITY trial investigators, Ashish Bhalla, Gregory Fox, Santosh Kumar Nag, Angela Makris, George Mangos, Jeffrey Post, Indu Ramachandra Rao, Louisa Sukkar, Richard Sullivan, Gian Luca Di Tanna, Jason Trubiano, Sophia Zoungas, Lovenish Bains, Atul Jindal, Nitin M Nagarkar, Saurabh Nayak

**Affiliations:** 1 NHMRC Clinical Trials Centre, University of Sydney, Camperdown, NSW, Australia; 2 Concord Repatriation General Hospital, Concord, NSW, Australia; 3 The George Institute for Global Health, University of New South Wales, Newtown, NSW, Australia; 4 Prince of Wales Hospital, Randwick, NSW, Australia; 5 The George Institute for Global Health, UNSW, New Delhi, India; 6 The George Institute for Global Health, Imperial College London, UK; 7 Sydney School of Public Health, University of Sydney, Camperdown, NSW, Australia; 8 Royal North Shore Hospital, St Leonards, NSW, Australia; 9 Kolling Institute of Medical Research, University of Sydney, St Leonards, NSW, Australia; 10 Department of Medicine, University of Melbourne, Austin Health, Heidelberg, VIC, Australia; 11 Institute of Breathing and Sleep, Heidelberg, VIC, Australia; 12 Queensland University of Technology, Brisbane, QLD, Australia; 13 All India Institute of Medical Sciences, Raipur, India; 14 Maulana Azad Medical College and Lok Nayak Hospital, New Delhi, India; 15 Jivanrekha Multispecialty Hospital, Pune, India; 16 Government Medical College and Hospital, Chandigarh, India; 17 Sydney Pharmacy School, The University of Sydney, Camperdown, NSW, Australia; 18 The Sydney Children’s Hospitals Network, Westmead, NSW, Australia; 19 Prasanna School of Public Health, Manipal Academy of Higher Education, Manipal, India; 20 School of Public Health, Imperial College, London, UK

## Abstract

**Objective:**

To determine whether disrupting the renin angiotensin system with angiotensin receptor blockers will improve clinical outcomes in people with covid-19.

**Design:**

CLARITY was a pragmatic, adaptive, multicentre, phase 3, randomised controlled trial.

**Setting:**

17 hospital sites in India and Australia.

**Participants:**

Participants were at least 18 years old, previously untreated with angiotensin receptor blockers, with a laboratory confirmed diagnosis of severe acute respiratory syndrome coronavirus-2 (SARS-CoV-2) infection who had been admitted to hospital for management of covid-19.

**Intervention:**

Oral angiotensin receptor blockers (telmisartan in India) or placebo (1:1) for 28 days.

**Main outcome measures:**

The primary endpoint was covid-19 disease severity using a modified World Health Organization Clinical Progression Scale (WHO scale) at day 14. Secondary outcomes were WHO scale scores at day 28, mortality, intensive care unit admission, and respiratory failure. Analyses were evaluated on an ordinal scale in the intention-to-treat population.

**Results:**

Between 3 May 2020 and 13 November 2021, 2930 people were screened for eligibility, with 393 randomly assigned to angiotensin receptor blockers (of which 388 (98.7%) to telmisartan 40 mg/day) and 394 to the control group. 787 participants were randomised: 778 (98.9%) from India and nine (1.1%) from Australia. The median WHO scale score at day 14 was 1 (interquartile range 1-1) in 384 participants assigned angiotensin receptor blockers and 1 (1-1) in 382 participants assigned placebo (adjusted odds ratio 1.51 (95% credible interval 1.02 to 2.23), probability of an odds ratio of >1 (Pr(OR>1)=0.98). WHO scale scores at day 28 showed little evidence of difference between groups (1.02 (0.55 to 1.87), Pr(OR>1)=0.53). The trial was stopped when a prespecified futility rule was met.

**Conclusions:**

In patients admitted to hospital for covid-19, mostly with mild disease, not requiring oxygen, no evidence of benefit, based on disease severity score, was found for treatment with angiotensin receptor blockers, using predominantly 40 mg/day of telmisartan.

**Trial registration:**

ClinicalTrials.gov NCT04394117.

## Introduction

The severe acute respiratory syndrome coronavirus-2 (SARS-CoV-2) virus enters host cells by binding to angiotensin-converting enzyme 2 (ACE2) receptors, triggering endocytosis.^1^ ^2^ ^3^ ^4^ ACE2, a key regulator of the renin angiotensin system, degrades angiotensin II and thus reduces its adverse effects.^4^ In preclinical mouse models of SARS-CoV (a related novel coronavirus, preceding SARS-CoV-2), binding to ACE2 led to dysregulation of the renin angiotensin system in local tissue, driving inflammation and fibrosis in the lung due to unopposed action of angiotensin II. These effects were reversed by angiotensin receptor blockers.^5^

Two studies showed that the Angiotensin II type 1 receptor (AT1R) is the crucial receptor that mediates vascular permeability and severe acute lung injury, induced by angiotensin II.^5^ ^6^ Pharmacological inhibition of AT1R attenuated the severity of acid induced lung injury in ACE2 knockout mice,^6^ and reduced pulmonary oedema and acute severe lung injury in a mouse model of SARS-CoV induced lung injury.^5^ These data suggested that modulation of the renin angiotensin system with ARBs might have protective effects in patients with SARS-CoV-2, and provided the rationale for our study.

No clear observational evidence about ARB use in humans in the covid-19 setting had been reported. Therefore, we conducted a randomised controlled trial featuring sample size adaptation, sequential analyses, and prespecified decision rules for futility and effectiveness, with an aim to establish evidence on the effect of ARB treatment on clinical progression in patients with SARS-CoV-2 infection.

## Methods

### Study design

CLARITY (Controlled evaLuation of Angiotensin Receptor blockers for covid-19 respIraTorY disease) was a pragmatic, adaptive, multicentre, phase 3, randomised, double blinded, controlled trial conducted at 17 sites in India and Australia. The protocol and statistical analysis plan have been published.^7^ ^8^

### Participants

Eligible participants were at least 18 years old with a laboratory confirmed diagnosis of SARS-CoV-2 infection, according to local standard operating procedures, who had been admitted to hospital for management of covid-19. Exclusion criteria included previous treatment with a renin angiotensin system blockade,^7^ high (>5.2 mmol/L) or unrecorded serum potassium, low (<30 mL per min/1.73 m^2^) estimated glomerular filtration rate, or unrecorded kidney function. The complete eligibility criteria and screening pathways are described in the protocol publication.^7^ Participants were screened at participating hospitals and covid-19 clinics. All participants provided witnessed written consent during clinical review or recorded verbal informed consent.

### Randomisation and masking

Participants were randomly assigned (1:1) to the ARB or control group by use of REDCap. The randomisation sequence was generated using Statistical Analysis Software (version 9.3), stratified by country, and had permuted blocks of size four and six. In India, the control arm was standard of care plus matched placebo. In Australia, for logistical reasons, the control arm was standard of care alone (no placebo), a limitation detailed in the protocol publication.^7^ The placebo tablets were identical in appearance, with identical packaging and labelling as the intervention. In India, the study was double blind; participants, study team, and clinicians were blinded to study group. In Australia, the clinical team assessing outcomes were unblinded, due to unavailability of placebo at an early stage of the pandemic. Data being analysed were unblinded to the trial statisticians by group assignment.

### Procedures

The intervention or control was prescribed once daily for 28 days. In India, treatment was with telmisartan at a starting dose of 40 mg or matched placebo. In Australia, choice of ARB was at treating physician discretion. In both countries, a low-to-moderate starting daily dose of 40 mg/day was recommended, with dose escalation permitted within the recommended dosing range for ARBs. The starting dose of 40 mg/day was selected because this dose is the approved Australian schedule for use of ARBs to treat hypertension, and therefore, a dose and safety profile that was familiar to clinicians. The study was designed as an effectiveness (not efficacy) study as a result of the well known properties of ARBs and the infectious nature of covid-19, with no in-person visits or follow-up procedures. Participating sites extracted trial data from medical records and obtained information by telephone after a patient was discharged from hospital.

### Outcomes

The trial was originally specified with a primary outcome of covid-19 disease severity measured using an ordinal scale with seven categories, from 1 (not admitted to hospital, no limitations on activities) to 7 (death), modified from the World Health Organization Clinical Progression Scale (WHO scale, appendix page 4),^9^ assessed at day 28. External evidence from other settings suggested that the WHO scale range was greater on day 14 than day 28, and therefore differences between treatment groups might be more apparent at day 14.^10^ In October 2020, the primary outcome was amended to a day 14 assessment of the WHO scale, and the assessment at day 28 was kept as a secondary outcome. None of the CLARITY trial data were reviewed before this change.

Other secondary outcomes were measured at day 28, including severity of covid-19 mortality, intensive care unit admission, and respiratory failure.

Prespecified safety endpoints were measured at day 28, including incidence of acute kidney injury (using kidney disease improving global outcomes guidelines^11^) and hypotension (vasopressor requirement). The exploratory endpoints included hyperkalaemia (serum potassium concentration of >6.0 mmol/L). Adverse events and serious adverse events were not collected beyond the prespecified outcomes because of the well established safety profile of ARBs.

### Statistical analysis

As a result of the broad range of plausible effect sizes of ARBs in patients with covid-19 and to efficiently manage resources, the trial incorporated an adaptive sample size component. This adaptive design allowed continued recruitment until prespecified decision thresholds for efficacy or futility were reached, or a maximum of 2200 participants were randomly assigned.

The primary intention-to-treat analysis used a bayesian inferential framework for all randomly assigned participants as outlined in the statistical analysis plan.^8^ We regressed the WHO scale at day 14 using an ordinal cumulative logistic regression model on treatment group assignment adjusted for age, sex, comorbidity, hypertension, and oxygen requirement at baseline. Interim analyses involved evaluating prespecified futility and success rules based on the results from the joint posterior and posterior predictive distributions.^8^ Sensitivity analyses included unadjusted models, relaxation of the proportional odds assumption, and comparison with analogous frequentist models. We sampled the joint posterior distribution using Markov Chain Monte Carlo. We imputed missing data using the standard Bayesian approach from the posterior predictive distribution. Subgroup analyses by age (<50 years or ≥50 years), sex, and oxygen requirement at baseline were performed, with treatment effects determined by subgroup. The trial is registered with ClinicalTrials.gov, number NCT04394117.

### Patient and public involvement

We convened a consumer and community engagement committee, as described within the protocol publication.^7^ This group assisted the trial steering committee, providing advice and feedback on trial aims, design, participant facing materials, and conduct. A lay language template for the results was developed and pre-approved by the relevant ethics committee to facilitate rapid dissemination of results to surviving participants at the conclusion of the trial.

## Results

**Fig 1:**
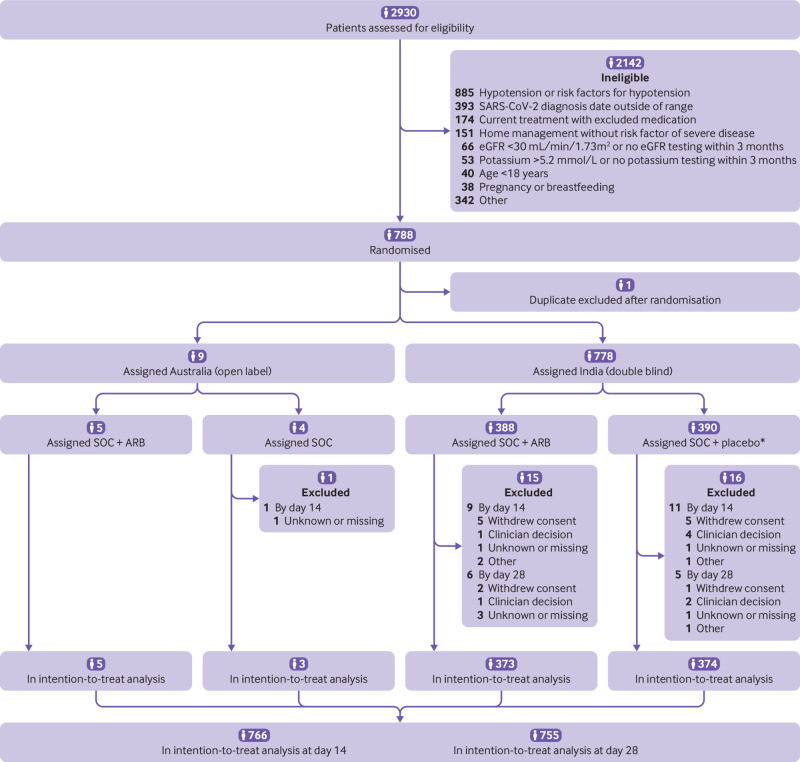
Participant flow in the CLARITY trial. eGFR=estimated glomerular filtration rate. SOC=standard of care. ARB=angiotensin receptor blocker. *Four did not receive allocated intervention

Between 3 May 2020 and 13 November 2021, 2930 patients were screened for eligibility. Overall, 788 participants were randomised. One participant was enrolled twice so the duplication was excluded after randomisation but before assignment. As such, of the 787 participants included, 778 (99%) participants were randomised from an Indian centre and nine (1%) from an Australian centre. For the intervention group, 393 (50%) people were assigned to ARB (of which, 388 (99%) were given telmisartan 40 mg/day) and 394 (50%) to the control group (fig 1). Treatment delivery was reported for all participants randomly assigned to ARB or the matching placebo (778). WHO scale scores were available for 766 (97%) participants on day 14 and for 755 (96%) participants on day 28. During the trial, 13 participants (2%) withdrew consent for continued treatment and use of subsequent data: seven in the ARB group and six in the control group.

The median age was 49 years (interquartile range 37-60), 505 (64%)were men, 282 (36%) were women, and 223 (28%) required supplemental oxygen at baseline (table 1). All participants, in India and Australia, were eligible based on being admitted to hospital for management of covid-19 disease. Nearly half (366 (47%)) had at least one comorbidity, whereas less than a third (232 (30%)) had a protocol defined comorbid risk factor for severe disease, most commonly were older than 60 years (189 (24%)), or had diabetes (197 (25%)). All participants in India identified as southern Asian. Prognostic factors were distributed evenly between the groups overall with small imbalances that did not systematically favour either group, with the exception that average C reactive protein values were slightly higher in the intervention group compared with the placebo. Worse disease in the ARB group would bias toward a null result. However, the difference is relatively small and unlikely to imply substantial difference in disease severity or to prompt altered management, and therefore was unlikely to impact study findings. At baseline, the median WHO scale score was 3 (interquartile range 3-4), with most participants 561 (71%) admitted to hospital and not requiring supplemental oxygen (score 3).

**Table 1:** Baseline characteristics of intention-to-treat population

	ARB group (n=393)	Control group (n=394)
Median (IQR) age (years)	49 (37-60)	49 (37-60)
Sex:		
Male	254 (65)	251 (64)
Female	139 (35)	143 (36)
Ethnic origin:		
Southern Asian	388 (99)	389 (99)
White	3 (1)	2 (1)
Other	2 (1)	3 (1)
Median (IQR) time between covid-19 diagnosis and randomisation (days)	3 (1-5)	3 (2-6)
Comorbidities:		
Chronic kidney disease	1 (<1)	5 (1)
Hypertension	93 (24)	122 (31)
Diabetes	90 (23)	107 (27)
Cardiovascular disease*	10 (3)	11 (3)
Cancer in past five years (excluding BCC/SCC)	2 (1)	0
Chronic respiratory illness	16 (4)	8 (2)
Obesity (BMI >30)	23 (6)	21 (5)
Age >60 years	96 (24)	93 (24)
Smoking status:		
Smokes	16 (4)	13 (3)
Previously smoked	60 (15)	59 (15)
Never smoked	317 (81)	322 (82)

Data are number (%) of participants unless stated otherwise. ARB=angiotensin receptor blocker; BCC=basal cell carcinoma; BMI=body mass index; IQR=interquartile range; SCC=squamous cell carcinoma.

* Including heart failure, ischaemic heart disease, acute myocardial infarction, congenital heart disease, stroke, peripheral vascular disease.

At the first planned analysis (conducted when 700 randomly assigned participants reached day 14 of follow-up), the data monitoring committee found that the prespecified criteria for futility were met, with predictive probability of trial success less than the prespecified decision threshold of 2%. The definitive analysis, which was based on 787 participants randomised and having reached the day 28 follow-up, confirmed the futility decision.

**Fig 2:**
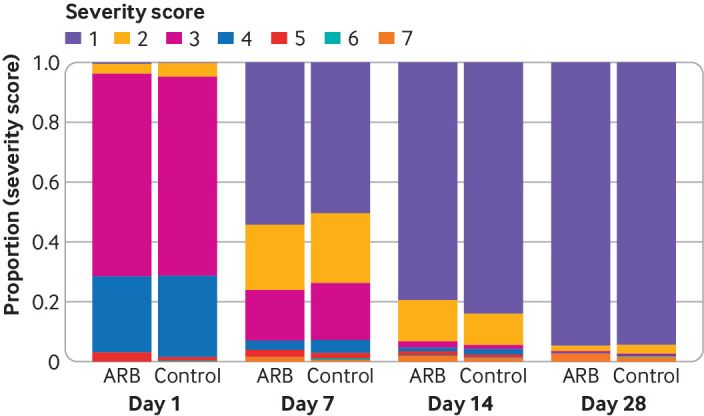
Bar graph showing WHO progression scale of covid-19 severity between study groups. ARB=angiotensin receptor blocker

**Fig 3:**
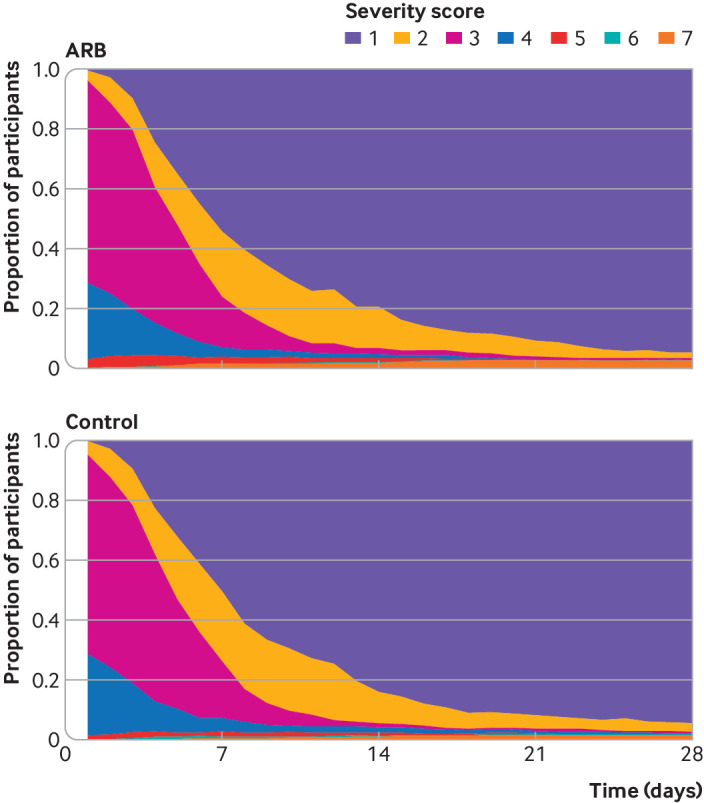
Line graph showing WHO progression scale of covid-19 severity between study groups. ARB=angiotensin receptor blocker

The overall median WHO scale score was 3 (in hospital not requiring oxygen) at baseline, which then improved to 1 (not admitted to hospital with no activity limitations) at day seven (interquartile range 1-3), 1 (1-1) at day 14, and 1 (1-1) day 28 (fig 2 and fig 3, appendix page 6). The median WHO scale score at day 14 was 1 (1-1) in 384 participants assigned angiotensin receptor blockers and 1 (1-1) in 382 participants assigned placebo.

Adjusted for baseline age, sex, protocol defined comorbidity, hypertension, and oxygen requirement, the primary outcome of WHO scale score at day 14 was worse in patients who received ARBs compared with control (adjusted odds ratio 1.51 (95% credible interval 1.02 to 2.23), probability of an odds ratio of >1 (Pr(OR>1))=0.98, fig 2 and fig 3). Results were similar in the sensitivity analyses that tested the effect of particular modelling assumptions.^6^ The unadjusted analysis (1.35 (0.94 to 1.95), Pr(OR>1)=0.94) and the analysis restricted to the placebo controlled cohort of participants (ie, participants recruited in India) (1.49 (1.00 to 2.23), Pr(OR>1)=0.98) gave numerically similar results (appendix page 9).

No evidence of heterogeneity of treatment effect was noted in any of the prespecified subgroups defined by age, sex, comorbidity, hypertension, or baseline oxygen requirement (appendix page 4).

The secondary outcome of adjusted WHO scale score at day 28 suggested negligible difference between treatment groups (odds ratio 1.02 (95% credible interval 0.55 to 1.87), Pr(OR>1)=0.53) (fig 2 and fig 3, appendix page 5). The proportion of participants with the best outcome (score 1; not admitted to hospital with no limitations) was higher in the ARB group at day seven (54.3% in the ARB group* v *50.4% in the control group), higher in the control group at day 14 (79.4% *v* 84%), and approximately the same in both groups at day 28 (95% *v* 94.4%) (fig 2 and fig 3, appendix page 6).

At 28 days, there were 16 deaths overall (10 in the ARB group, six in the control group; odds ratio 1.74 (95% credible interval 0.62 to 5.25), Pr(OR>1)=0.85). No differences were observed between groups for the prespecified safety outcomes of acute kidney injury, hyperkalaemia, or hypotension, or for other secondary and exploratory outcomes, noting that event numbers for some of these outcomes were low (table 2).

**Table 2:** Secondary outcomes

	ARB group (n=393)				Control group (n=394)			Measurement (odds ratio or hazard ratio†) (95% CrI)	Probability of odds ratio or hazard ratio >1†
	No with event	No without event	Total No*		No with event	No without event	Total No*		
**Dichotomous and ordinal outcomes (odds ratios)**									
Mortality‡	10	368	378		6	371	377	1.74 (0.62 to 5.25)	0.85
WHO scale at day 28§	NA	NA	378		NA	NA	377	0.97 (0.53 to 1.76)	0.46
**Time to event analyses (hazard ratios)**									
Discharge alive	374	16	390		381	13	394	1 (0.87 to 1.15)	0.52
Intensive care unit admission	26	364	390		32	362	394	0.79 (0.47 to 1.35)	0.19
Respiratory failure	26	367	393		20	374	394	1.32 (0.74 to 2.43)	0.83
Dialysis	1	367	378		2	369	377	Not estimated**	NA
Acute kidney injury	30	363	393		26	368	394	1.15 (0.67 to 1.96)	0.69
Hyperkalaemia	2	121	123		0	124	124	Not estimated	NA
Hypotension	7	386	393		4	390	394	Not estimated	NA

ARB=angiotensin receptor blocker. CrI=credible interval. NA=not available.

* Number informing the posterior distribution; this varied based on availability of variables necessary for the analysis.

† Parameter relates to treatment term in linear predictor (unadjusted model).

‡ Dichotomous outcome.

§ Ordinal outcome.

¶ Including at trial entry, defined as invasive mechanical ventilation, extracorporeal membrane oxygenation, non-invasive ventilation, or high flow oxygen during hospital stay.

** Treatment effects were not estimated when the total number of events was fewer than 20.

## Discussion

### Principal findings

Patients admitted to hospital for covid-19 were randomly assigned to an ARB (predominantly telmisartan) or control for 28 days, with no benefit in the primary outcome of clinical severity as assessed by the WHO scale at day 14. In fact, the primary outcome showed a 98% probability of an increase in the odds of higher severity scores at day 14 for the use of ARB compared with control. However, the effect was not temporally consistent. The results of day seven and 14 were in opposing directions and the day 28 results suggested no clear difference between the groups. Overall, the differences between results at days seven, 14, and 28 are not considered to be clinically meaningful. ARBs, as used in CLARITY, were not associated with an increase in the nominated adverse effects of recorded acute kidney injury, hypotension, and hyperkalaemia, although these effects were mitigated by trial entry criteria that excluded patients at higher risk of these conditions. With confidence, we conclude that, contrary to the trial hypothesis, ARBs (that were mostly administered as 40 mg/day of telmisartan) had no benefit for clinical outcomes assessed by the WHO scale in this population.

Although the effect of ARBs on days seven and 14 seems to be opposing, the actual differences in the groups were driven by transient differences at the mild end of the scale in the proportion reporting some or no limitations while at home. The nature of the score means these differences contribute as much to the results as a one-step difference at the severe end of the score; for example, the difference between requiring high flow nasal cannula oxygen or being intubated, or the difference between being intubated and death. The harm is mild and transient, is balanced by a similarly transient benefit at day seven, and did not prove robust to sensitivity testing. While a plausible causal basis for a reversal in treatment effects could be postulated, the heterogeneity in effects likely represents chance variation around an overall null effect. The trial outcomes highlight the limitations of evaluating a therapeutic intervention at a fixed point in time when the response is inherently temporal and multidimensionally complex.

Our trial cohort had milder disease than anticipated, with nearly 75% of the participants having returned home by day seven and more than 90% of participants had returned home by day 14. This effect left a relatively sparse distribution of participants admitted to hospital, which should be considered when interpreting the results and determining their clinical relevance. Overall, participants were younger than in previous reports, and thus, at lower risk of severe outcomes.^12^ ^13^ ^14^ In fact, we observed low numbers of events for some prespecified secondary outcomes (eg, dialysis requirement) that precluded informative analysis.

Most participants were from India and of southern Asian ethnicity, a population reported to be at increased risk of poor covid‐19 outcomes.^15^ A benefit for other ethnicities cannot be ruled out; however, ARB activity as an antihypertensive is not reported to vary by ethnicity. Few participants required supplemental oxygen at recruitment (28%), again suggesting relatively mild disease in our study rather than reduced access to treatment. All participating sites in India had an oxygen generator and no sites in Australia had supply limitations. A fifth of the cohort were receiving systemic steroids at recruitment, an intervention shown to reduce mortality, most clearly in people requiring oxygen or mechanical ventilation at baseline.^16^ CLARITY participants had substantially better outcomes than participants in trials conducted early in the pandemic, largely in Europe, the UK, and the USA. The overall mortality rate of 2% in the first 28 days was lower than reports of 8% or higher early in the pandemic (October 2020) and was consistent with falling case fatality rates during the study (3.5% at start of study, 2.1% at close of recruitment, and 1.5% at time of writing).^17^ The average health status of CLARITY participants was worst at the time of recruitment, after which their health improved, in contrast with earlier studies that showed worsening health status, with the widest range of scores apparent at day 14.^18^ ^19^ Therefore, the generalisability of the results might be limited to younger patients with low severity disease and treated with 40 mg/day telmisartan. We cannot exclude a benefit or harm from ARBs in patients with more severe disease or treated with different agents or dosing.

Precedent evidence supports the concept that interventions might have differential effects in participants with different risk of covid-19 severity. The RECOVERY trial showed that corticosteroids are effective overall for patients with covid-19 admitted to hospital but are not beneficial in patients at lower risk who did not require respiratory support at enrolment.^16^ The role of ARBs in more severe disease is being investigated in the ongoing platform REMAP-CAP trial, which is recruiting participants with covid-19 admitted to critical care units.^20^

### Strengths and limitations of this trial

Strengths of the trial included its adaptive sample size, which enabled a test of both efficacy and futility without a definitive prior estimate of effect size. The careful simulation planning showed a design that appeared robust to foreseen and unforeseen variations in control outcomes, albeit with differing interpretation.^8^ The planned analyses meant that the trial did not continue beyond futility, hence limiting expenditure and resourcing on a therapy that indicates no benefit in the trial population. The use of modified endpoints sourced from WHO facilitated comparison with other covid-19 trials.^9^ Adherence to study treatment and follow-up for the 28 day intervention period was good. The trial deliberately used a wide geographical spread of sites in India and Australia to facilitate recruitment despite the unpredictability of covid-19 caseloads.^7^ This design allowed effectiveness to be assessed under real-world conditions; however, 99% of our recruitment was from India because of the trial being conducted while India had a major increase in number of covid-19 cases caused by the delta variant (B.1.617.2) of SARS-CoV-2. 

Limitations of the current study include the inability to source placebo in Australia, meaning the study was not blinded to participants or treating clinicians. However, the absence of placebo probably had minimal impact because only nine participants were recruited to the open label component of the study. Despite the use of placebo in India, the treatment profile of ARBs is well known, which could potentially have unblinded treating clinicians. In our study, participants were treated with a relatively low dose of 40 mg telmisartan, and the effect of higher doses remains unknown. The trial did not collect all safety events because of the well established safety profile of ARBs, and even the maximum anticipated sample size would be insufficient to establish whether the safety profile in the covid-19 setting was significantly different from that in non-covid-19 settings. As CLARITY was a pragmatic trial, clinical investigations for potential adverse events were initiated at the treating clinicians discretion, meaning subclinical adverse events might not have been detected, even though information on known safety events were collected throughout.

### Comparison with other studies

The finding that ACE2 mediates SARS-CoV-2 cell entry, combined with clinical equipoise on the use of renin angiotensin system blockade for the management of covid-19 early in the pandemic, led to two groups of randomised trials internationally: those that randomly assigned pre-existing users of renin angiotensin system blockade to cessation; and those that randomly assigned patients who were previously untreated with renin angiotensin system blockade to renin angiotensin system blockade. The findings from CLARITY are largely consistent with five previous trials in which 593 participants previously untreated with a renin angiotensin system inhibitor were randomly assigned to receive treatment with an ARB.^21^ ^22^ ^23^ ^24^ ^25^ Four trials, which randomly assigned 435 participants, reported no effect on the primary outcomes of 30 day mortality,^23^ length of hospital stay,^23^ a composite of mechanical ventilation or death,^22^ lung injury,^24^ and all cause hospital admission (among patients not admitted to hospital).^25^ Additionally, no effect was reported for most of the secondary outcomes examined. These findings were true for a range of dosing schedules, ranging from 12.5 mg twice a day for 10 days^22^ to 50 mg twice a day for the same duration.^24^ In an unblinded trial of 158 patients who were given high dose telmisartan (80 mg twice a day for 14 days) or standard care, C reactive protein plasma concentrations were lower in the telmisartan group, with a mean value of 3.83 at day five and 2.37 at day eight compared with the control group who had 6.06 at day five and 6.30 at day eight.^21^ The trial also reported benefit for a range of secondary outcomes, including a reduced risk of death at days 15 and 30 and reduced risk of intensive care unit admission. Differences in the two studies included the younger mean age of the CLARITY cohort (49 years *v* 65 years), the lower dose of telmisartan used (daily dose of 40 mg *v* 160 mg), and the potential for inclusion later in disease course in CLARITY (up to 10 days after diagnosis compared with up to four days since symptom onset in the trial by Duarte et al). 21 Other differences included that the trial by Duarte et al was unblinded, that the number of secondary events in both studies was low (19 deaths and 21 intensive care unit admissions), and that the trial was terminated earlier than planned due to a sharp drop in recruitment. Although our protocol allowed dose escalation if tolerated, very few participants were given doses above 40 mg/day, perhaps reflecting a cautious approach by clinicians warranted by the limited experience with higher doses.

Four randomised trials of cessation, of 1061 participants, reported no difference between patients who continued renin angiotensin system inhibitors and those who stopped for a range of outcomes and in different patient populations.^18^ ^19^ ^26^ ^27^ The finding of a neutral effect of renin angiotensin system inhibitor discontinuation across the four trials, which differed in size, location, and patient characteristics, supports the safe continuation of renin angiotensin system blockade in patients already receiving these medications. Another factor potentially affecting ARB activity is timing; for example, if clinical efficacy of ARB is attenuated by a delay between initiation of the inflammatory process by infection with SARS-CoV-2 and the start of ARB administration, or if treatment with ARB is required for a period before infection to see an effect. The participants in CLARITY were randomised relatively early in their disease course (median three days since diagnosis), which seems similar to other trials,^18^ ^19^ ^20^ ^21^ ^22^ ^24^ ^25^ ^26^ ^27^ although variations in reporting make a direct comparison difficult. These hypotheses might be challenging to test, but nevertheless, they might be an important reason as to why benefit for ARB was not observed. Overall, evidence from CLARITY and previous, smaller trials suggests that ARBs are safe to use in covid-19 but are unlikely to be beneficial, particularly in the context of other effective medications.

### Conclusions

In patients predominantly from southern Asian who were admitted to hospital for covid-19, but mostly not requiring oxygen support at baseline, ARBs that were predominantly administered as 40 mg/day of telmisartan were not an effective treatment to reduce disease severity at day 14. Ongoing trials might assess the effect of ARBs in more severe disease. The use of an adaptive bayesian design ensured the question was definitively and efficiently answered, to inform clinical practice even in the setting of a novel disease.

**What is already known on this topic**

ACE2, a component of the renin angiotensin system, mediates SARS-CoV-2 entry into cells, leading researchers to explore the role of renin angiotensin system blockade (ARB) in covid-19
Existing clinical trials have focused on either randomisation of people who used renin angiotensin system inhibitors until drug cessation, or randomisation of those who had never used a renin angiotensin system blockade to this blockade
Five trials of 593 ARB untreated participants, randomised to renin angiotensin system inhibitors also reported neutral effects on primary outcomes, although one trial reported a benefit of high dose telmisartan (80 mg twice daily for 14 days) for the secondary outcome of mortality based on 19 deaths overall

**What this study adds**

With 787 participants, CLARITY reported no benefit of ARBs, predominantly 40 mg/day of telmisartan, in people admitted to hospital for covid-19 with low disease severity, based on WHO disease severity score at day 14
This bayesian adaptive sample size design ensured that ARB use for covid-19 was addressed in a timely manner with sufficient power while minimising resource use, highlighting the role for adaptive trials to accelerate evidence generation

## Data Availability

The final dataset will be under the custodianship of the chair of the trial steering committee. all individual participant data that are collected during the trial will be de-identified. The researchers intend de-identified data will contribute to global learnings through an appropriately constituted individual participant data level collaboration. Requests for data access or analysis proposals will be reviewed by the trial steering committee, who will assess proposals according to criteria based on scientific merit and contribution to global knowledge. Data sharing will be performed in compliance with local data protection laws.

## Web extra

Extra material supplied by authors

Web appendix: Supplementary material

## Notes

Members of the CLARITY trial investigators are listed in the appendix (pages 1-3).

## Notes

Contributors: MJJ contributed to conceptualisation, data curation, funding acquisition, investigation, methodology, project administration, resources, software, supervision, visualisation, writing the original draft, and writing, reviewing and editing, and is a guarantor of the study. SSK contributed to data curation, funding acquisition, investigation, methodology, project administration, writing the original draft, and reviewing and editing. AB contributed to data curation, investigation, methodology, project administration, resources, software, writing the original draft, and reviewing and editing. CH contributed to data curation, investigation, methodology, project administration, writing the original draft, and reviewing and editing. MJ contributed to formal analysis, methodology, software, visualisation, writing the original draft, and reviewing and editing. AW contributed to data curation, investigation, methodology, project administration, resources, software, writing the original draft, and reviewing and editing. CP contributed to data curation, funding acquisition, investigation, methodology, and writing, and reviewing and editing. LMB contributed to data curation, funding acquisition, investigation, methodology, writing, and reviewing and editing. JM contributed to formal analysis, funding acquisition, methodology, software, visualisation, writing the original draft, and reviewing and editing. VR contributed to investigation, resources, and writing, and reviewing and editing. CRJ contributed to data curation, funding acquisition, investigation, and writing, and reviewing and editing. LG contributed to investigation, resources, and writing, and reviewing and editing. AR contributed to data curation, funding acquisition, investigation, resources, writing, and reviewing and editing. AB and SDC contributed to investigation, resources, and writing, and reviewing and editing. AJM contributed to funding acquisition, methodology, and writing, and reviewing and editing. SF contributed to funding acquisition, and writing, and reviewing and editing. MMC contributed to project administration, visualisation, writing the original draft, and reviewing and editing. TS contributed to formal analysis, methodology, resources, software, visualisation, and writing, and reviewing and editing. VJ contributed to data curation, investigation, methodology, project administration, resources, supervision, writing the original draft, reviewing and editing, and is a guarantor of the study. The corresponding author attests that all listed authors meet authorship criteria and that no others meeting the criteria have been omitted.

The lead authors (MJJ and VJ) affirm that the manuscript is an honest, accurate, and transparent account of the study being reported; that no important aspects of the study have been omitted; and that any discrepancies from the study as originally planned have been explained.

Dissemination to participants and related patient and public communities: The study convened a consumer and community engagement committee as described within the protocol publication.^7^ This group provided advice and feedback on the template for trial results dissemination, which was pre-approved by the ethics committee to facilitate dissemination of results to surviving participants at the conclusion of the trial. Additionally, the results of the trial will be disseminated through the consumer and community engagement committee, presentation at conferences and other appropriate forums, through relevant social media channels, and a plain English summary on the sponsors website.

Provenance and peer review: Not commissioned; externally peer reviewed.
