# Supplementary material for: Angiotensin receptor blockers for the treatment of covid-19: pragmatic, adaptive, multicentre, phase 3, randomised controlled trial
Source: BMJ. 2022 Nov 16;379:e072175. doi: 10.1136/bmj-2022-072175 (PMC9667467; doi:10.1136/bmj-2022-072175)
Supplement: Supplementary file 1 — Web appendix: Supplementary material [file jarm072175.ww.pdf]

Supplementary Appendix

Contents

1. CLARITY Trial Investigators 1
2. Data Safety Monitoring Board 2
3. Collaborating Sites and Investigators 2
4. Operational Teams 4
5. Protocol 4
6. Subgroup Analysis of Primary Outcome 4
7. Inclusion/exclusion Criteria 5
   - Inclusion Criteria at Screening Visit: 5
   - Exclusion Criteria: 5
8. Supplementary Table S1. Modified WHO Clinical Progression Scale 6
9. Supplementary Table S2. Additional Demographics and Clinical Characteristics 6
10. Supplementary Table S3. Treatment dose, Concomitant Medications, and Trial Discontinuation to day 28 7
11. Supplementary Table S4. Model Sensitivity Results 10
12. Reference 10

1. CLARITY Trial Investigators

Steering Committee Executive: Meg Jardine (Chair), Vivekanand Jha (Co-Chair), Abhinav Bassi, Louise Burrell, Carinna Hockham, Christine Jenkins, Sradha Kotwal, Carol Pollock, Angus Ritchie, Arlen Wilcox.

Steering Committee Members: Ashpak Bangi, Ashish Bhalla, Sanjay D’Cruz, Simon Finfer, Gregory Fox, Lalit Gupta, Santosh Kumar Nag, Mark Jones, Angela Makris, George Mangos, James McGree, Andrew McLachlan, Jeffrey Post, Vinay Rathore, Indu Ramachandra Rao, Thomas Snelling, Louisa Sukkar, Richard Sullivan, Gian Luca Di Tanna, Jason Trubiano, Sophia Zoungas.

India Regional Committee: Vivekanand Jha (Chair), Abhinav Bassi (Co-Chair), Lovenish Bains, Ashpak Bangi, Ashish Bhalla, Sanjay D'Cruz, Lalit Gupta, Atul Jindal, Santosh Kumar Nag, Nitin M. Nagarkar, Saurabh Nayak, Vinay Rathore, Indu Ramachandra Rao.

Writing committee: Meg J. Jardine PhD[1,2], Sradha S. Kotwal PhD[3,4], Abhinav Bassi MPH[5], Carinna Hockham DPhil[6], Mark Jones MBiostat[7], Arlen Wilcox BBiomedSc[1], Carol Pollock PhD[8,9], Louise M. Burrell MD[10,11], James McGree PhD[12], Vinay Rathore MBBS[13], Christine R. Jenkins MD[2,3], Lalit Gupta DNB[14], Angus Ritchie MBBS[2], Ashpak Bangi MD[15], Sanjay D'Cruz MD[16], Andrew McLachlan PhD[7], Simon Finfer MD[3], Michelle M. Cummins PhD[1], Thomas Snelling PhD[7,17], Vivekanand Jha MD[5,18,19]. The members of the writing committee assume responsibility for the overall content and integrity of this article.

2. Data Safety Monitoring Board

Katherine Tuttle (Chair), Jonathan Craig, Stephane Heritier, Allison Lambert

3. Collaborating Sites and Investigators

Site	Location	Principal Investigator	Co-Investigator (Co-I) / Study Coordinator (SC)
**All India Institute of Medical Science, Raipur**	Chhattisgarh, India	Vinay Rathore	Saurabh Nayak (Co-I)<br>Atul Jindal (Co-I)<br>Nitin M Nagarkar (Co-I)<br>Ajoy Behera (Co-I)<br>MD Sabah Siddiqui (Co-I)<br>Rajendra Kumar Sahu (SC)<br>Anjulata Sahu (SC)
**Maulana Azad Medical College and Lok Nayak Hospital**	New Delhi, India	Lalit Gupta	Lovenish Bains (Co-I)<br>Manisha Dattatray Bhandwalker (SC)<br>Sushmita Gupta (SC)

**Jivanrekha Multispecialty Hospital**	Pune, India	Ashpak Bangi	Yasmeen Shaikh (Co-I)
**Government Medical College and Hospital**	Chandigarh, India	Sanjay D'Cruz	Yuvraj Singh Cheema (Co-I)<br>Mehak Trehan (SC)<br>Sahil Gupta (SC)
**Christian Hospital, Nabarangpur**	Odisha, India	Santosh Kumar Nag	Michael John (Co-I)
**Postgraduate Institute of Medical Education & Research**	Chandigarh, India	Ashish Bhalla	Deepak Sharma (SC)
**Kasturba Medical College, Manipal**	Karnataka, India	Indu Ramachandra Rao	Afsal PM (Co-I)
**Royal Prince Alfred Hospital**	New South Wales (NSW), Australia	Gregory Fox	Nuria Zamora (SC)
**Royal North Shore Hospital**	NSW, Australia	Carol Pollock	Muh Geot Wong (Co-I)<br>Dawn Ngai (SC)<br>Helen Clayton (SC)<br>Martyn Ralph (SC)
**Austin Hospital**	Victoria, Australia	Jason Trubiano	Louise Burrell (Co-I)<br>Fiona James (SC)
**St George Hospital**	NSW, Australia	Richard Sullivan	Sharon Robinson (SC)
**Royal North Shore Hospital**	NSW, Australia	Carol Pollock	Dawn Ngai (SC)<br>Helen Clayton (SC)<br>Martyn Ralph (SC)
**Concord Repatriation General Hospital**	NSW, Australia	Angus Ritchie	Samantha Hand (SC)<br>Yennie Huynh (SC)
**Liverpool Hospital**	NSW, Australia	Angela Makris	Deepa Francis (SC)
**Westmead Hospital**	NSW, Australia	Matthew O'Sullivan	Neela Joshi (Sc)
**Prince of Wales Hospital**	NSW, Australia	Jeffrey Post	Kristen Overton (SC)

**Wollongong Hospital**	NSW, Australia	Jenny Chen	Yulan Shen (SC)

4. Operational Teams

Study Clinician: Sradha Kotwal

Scientific Leads: Abhinav Bassi, Carinna Hockham, Sradha Kotwal

Project Manager: Arlen Wilcox

Central Management Team: Grace Balicki, Kirston Barton, Nikita Bathla, Alison Coenen, Michelle Cummins, Sedricx Fontanilla, Enmoore Lin, Martyn Ralph, Nuria Zamora, Shaz Sazali

Statistical Team: James McGree, Mark Jones, Tom Snelling, Michael Dymock

5. Protocol

The trial protocol has been published and is available online at the following location:

https://trialsjournal.biomedcentral.com/articles/10.1186/s13063-021-05521-0

6. Subgroup Analysis of Primary Outcome

Subgroup comparison	*Participants (n)* ARB	Control	*Log Odds Ratio (95% CI)*	*OR (95% CI)*
Age				
<50 years	198	195		1.54 (0.94, 2.56)
≥50 years	186	187		1.47 (0.90, 2.33)
Sex				
Female	136	140		1.39 (0.75, 2.29)
Male	248	242		1.58 (1.02, 2.52)
Comorbidities				
Absent	279	265		1.54 (0.99, 2.44)
Present	105	117		1.43 (0.79, 2.35)
Hypertension				
Absent	297	264		1.44 (0.88, 2.23)
Present	87	118		1.70 (1.03, 3.34)
Oxygen requirement				
No requirement	279	276		1.50 (0.94, 2.38)
Requirement	105	106		1.53 (0.96, 2.47)
Overall	384	382		1.51 (1.02, 2.23)

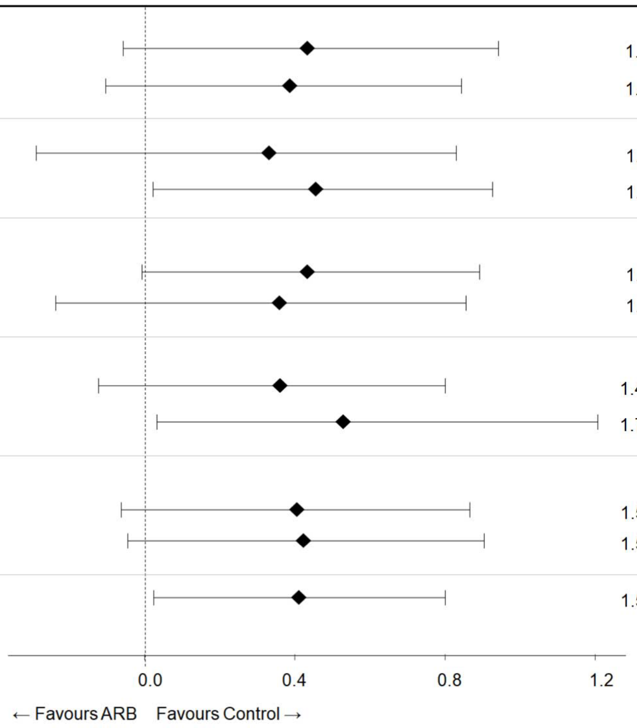

7. Inclusion/exclusion Criteria

Inclusion Criteria at Screening Visit:

Potential patients must satisfy all criteria:

1. Laboratory-confirmed* diagnosis of SARS-CoV-2 infection within 10 days prior to randomization

2. Age ≥ 18 years

3. a) Systolic Blood Pressure ≥ 120 mmHg

OR

b) SBP ≥ 115 mmHg and currently treated with a non-RAASi Blood Pressure lowering agent that can be ceased

4. Participant and treating clinician are willing and able to perform trial procedures.

5. Intended for hospital admission for management of COVID-19, *OR* (in Australia only) intended for management at home with one or more of the following criteria:

a. Age ≥ 60 years

b. BMI ≥ 30kg/m^2 (derived from the patient's self-report of their height and weight where these are not measured directly)

c. Diagnosis of diabetes defined as HbA1c ≥7% and/or the consumption of glucose lowering medication

d. History of cardiovascular disease

e. History of chronic respiratory illness

f. Currently treated with immunosuppression

*Confirmation through appropriate approved laboratory or Point of Care testing method, including Polymerase Chain Reaction (PCR) or other public health assay.

Exclusion Criteria:

Potential patients must not meet any of the following:

1. Currently treated with an ACEi, ARB or aldosterone antagonist, aliskiren, or angiotensin receptor-neprilysin inhibitors (ARNi)

2. Serum potassium > 5.2 mmol/L or no potassium testing within the last 3 months

3. For those intended for hospital admission, an estimated Glomerular Filtration Rate (eGFR) < 30ml/min/1.73m^2 or no eGFR testing within the last 3 months OR, for those intended for management at home (Australia only), an eGFR < 45ml/min/1.73m^2 or no eGFR testing within the last 3 months

4. Known symptomatic postural hypotension

5. Known biliary obstruction, known severe hepatic impairment (Child-Pugh-Turcotte score[1] 10-15).

6. Intolerance of ARB

7. Pregnancy or risk of pregnancy, defined as; a. (In Australia only) Women younger than 51 years who have not had a negative pregnancy test during the past 3 days and/or who do not agree to use adequate contraception b. (In India Only) Women who are pregnant

8. Women who are currently breastfeeding

9. Individuals who are not able to take medications by mouth at enrolment, or who are not expected to be able to take medications by mouth during the first 48 hours after randomization

Co-enrolment in other randomized controlled trials not involving RAS blockade treatment is encouraged.

8. Supplementary Table S1. Modified WHO Clinical Progression Scale

1	Not hospitalized, no limitations on activities
2	Not hospitalized, some limitation on activities
3	Hospitalized, not requiring supplemental oxygen
4	Hospitalized, requiring supplemental oxygen
5	Hospitalized, requiring non-invasive mechanical ventilation or high flow nasal cannula therapy
6	Hospitalized, requiring invasive mechanical ventilation +/- additional organ support
7	Death

9. Supplementary Table S2. Additional Demographics and Clinical Characteristics

	**ARB**	**Control**	**Total**
**Physical Examination –** mean (SD)	(N=393)	(N=394)	(N=787)
Systolic Blood pressure, mmHg	133.6 (9.9)	133.0 (9.2)	133.3 (9.50
Diastolic blood pressure, mmHg	83.4 (7.4)	83.5 (6.9)	83.5 (7.2)
	n=15	n=21	n=36

Oxygen saturation[1] - %	93.7 (1.7)	94.5 (1.7)	94.2 (1.7)
	n=68	n=79	n=147
BMI, kg/m^2	24.0 (3.6)	24.1 (3.5)	24.1 (3.6)
**BMI category (WHO) - n (%)**			
Underweight (BMI <18.5)	10 (2.5%)	8 (2.0%)	18 (2.3%)
Normal weight (BMI 18.5 - <25)	236 (60.1%)	226 (57.4%)	462 (58.7%)
Pre-obesity (BMI 25 - <30)	124 (31.6%)	138 (35.0%)	262 (33.3%)
Obesity class I (BMI 30 - <35)	16 (4.1%)	16 (4.1%)	32 (4.1%)
Obesity class II (BMI 35 - <40)	5 (1.3%)	3 (0.8%)	8 (1.0%)
Obesity class III (BMI ≥ 40)	2 (0.5%)	2 (0.5%)	4 (0.5%)
**Laboratory Measures –** mean (SD)	n=393	n=394	n=787
Serum Potassium, mmol/ l	4.0 (0.5)	4.1 (0.5)	4.1 (0.5)
CKD-Epi eGFR[2] ml/min/1.73 m^2	93.8 (37.9)	93.0 (24.1)	93.4 (31.7)
White Cell Count, x 10^9/ml	7.4 (3.5)	7.4 (4.0)	7.4 (3.8)
	n=185	n=185	n=370
C-Reactive Protein, mg/l	85.2 (167.3)	74.7 (133.9)	79.9 (151.4)

1. Only reported for those requiring supplemental oxygen

2. eGFR - estimated glomerular filtration rate

10. **Supplementary Table S3.** ***Treatment dose, Concomitant Medications, and Trial Discontinuation to day 28***

Treatment administration, adherence
Administration and adherence to IMP (ARB)

	Randomised to ARB	Received at least one dose of study treatment No. (%)	Days[2] - med (min to max, IQR)	Mean dose per day - mg (SD)
India	388	388 (100)	28 (1 to 28, 1)	40.1 (1.5)
Australia	5	5 (100)	27 (4 to 28, 1)	Not estimated

Concomitant medication[1]						
	At baseline			During follow up		
	ARB N = 393	Control N = 394	Total N = 787	ARB N = 393	Control N = 394	Total N = 787
Open-label RAS (ACEi, ARB, Aldosterone antag, ARNi, Aliskiren)	0.3	0.3	0.3	0.3[2]	0	0.1
Non-RASi Blood Pressure (BP) lowering agent	14	21	17	6.1	10	8.0
Oral/IV steroids	22	23	23	26	29	27
Inhaled steroids	<1.0	<1.0	<1.0	<1.0	<1.0	<1.0
Immunosuppressant therapies	<1.0	1.0	<1.0	<1.0	<1.0	<1.0
Antimicrobials	43	44	44	52	50	51
COVID-19 directed therapies:						
Remdesivir	6.9	10	8.6	8.4	12	10
Hydroxychloroquine	1.0	0	<1.0	1.0	0	<1.0
Favipiravir	<1.0	<1.0	<1.0	<1.0	<1.0	<1.0
Ivermectin	<1.0	0	<1.0	<1.0	0	<1.0

Convalescent plasma	0	0	0	0	<1.0	<1.0
Enoxaparin	<1.0	1.8	1.1	1.0	2.3	1.7
Multivitamin	0	<1.0	<1.0	0	<1.0	<1.0

1. Participants may have taken more than one medication at baseline and during follow up.

2. Open-label use of ARB in India reported

11. Supplementary Table S*4. Model sensitivity results*

The following table presents the results from the primary analysis on: a) complete data set (Primary analysis model), b) reduced linear predictor (Reduced primary model), c) placebo controlled cohort only, d) adjacent category model for assessment of proportional odds, and e) frequentist (Generalised Linear Model).

Term	Model				
	Primary analysis model OR 95% CrI	Reduced primary model OR 95% CrI	Primary analysis model – placebo controlled cohort OR 95% CrI	Adjacent categories model OR 95% CrI	Generalised Linear Model OR 95% CI
Treatment (ARB)	1.51 (1.02 to 2.23)	1.35 (0.94 to 1.95)	1.49 (1 to 2.23)		1.53 (1.04 to 2.28)
Category 1 vs 2*				1.39 (0.91 to 2.15)	
Category 2 vs 3*				1.14 (0.35 to 3.86)	
Category 3 vs 4*				0.48 (0.1 to 2.36)	
Category 4 vs 5*				1.78 (0.29 to 10.8)	

Category 5 vs 6*				0.44 (0.01 to 8.31)	
Category 6 vs 7*				3.44 (0.19 to 126.6)	
Age (>=50 years)	1.3 (0.86 to 1.96)		1.33 (0.88 to 2.03)		1.31 (0.86 to 2)
Sex (male)	1.27 (0.85 to 1.9)		1.23 (0.81 to 1.87)		1.27 (0.85 to 1.93)
Comorbidities (present)	1.48 (0.98 to 2.24)		1.43 (0.94 to 2.19)		1.5 (0.98 to 2.26)
Hypertension (present)	1.57 (1.03 to 2.37)		1.62 (1.05 to 2.47)		1.6 (1.05 to 2.43)
Oxygen requirement (modified WHO clinical progression score >=4)	4.43 (3.00 to 6.55)		4.50 (3.09 to 6.71)		4.62 (3.12 to 6.89)

* Category refers to the modified WHO clinical progression scale, e.g. Category 1 vs 2 refers to modified WHO clinical progression score <=1 vs modified WHO clinical progression score >=2.

12. Reference

1. Hockham C, Kotwal S, Wilcox A, et al. Protocol for the Controlled evaLuation of Angiotensin Receptor blockers for COVID-19 respIraTorY disease (CLARITY): a randomised controlled trial. Trials 2021; 22:573.
